# COPD exacerbation: Lost in translation

**DOI:** 10.1186/1471-2466-9-6

**Published:** 2009-01-29

**Authors:** Demosthenes Makris, Demosthenes Bouros

**Affiliations:** 1 Department of Pneumonology, Medical School, Democritus University of Thrace, Alexandroupolis, Greece

## Abstract

The introduction and acceptance of a standard definition for exacerbations of COPD can be helpful in prompt diagnosis and management of these events. The latest GOLD executive committee recognised this necessity and it has now included a definition of exacerbation in the guidelines for COPD which is an important step forward in the management of the disease. This definition is pragmatic and compromises the different approaches for exacerbation. However, the inclusion of the "healthcare utilisation" approach (".. may warrant a change in regular medication") in the definition may introduce in the diagnosis of exacerbation factors related to the access to health care services which may not be related to the underlying pathophysiologal process which characterizes exacerbations. It should be also noted that the aetiology of COPD exacerbations has not yet been included in the current definition. In this respect, the definition does not acknowledge the fact that many patients with COPD may suffer from additional conditions (i.e. congestive cardiac failure or pulmonary embolism) that can masquerade as exacerbations but they should not be considered as causes of them. The authors therefore suggest that an inclusion of the etiologic factors of COPD exacerbations in the definition. Moreover, COPD exacerbations are characterized by increased airway and systemic inflammation and significant deterioration in lung fuction. These fundamental aspects should be accounted in diagnosis/definition of exacerbations. This could be done by the introduction of a "laboratory" marker in the diagnosis of these acute events. The authors acknowledge that the use of a test or a biomarker in the diagnosis of exacerbations meets certain difficulties related to performing lung function tests or to sampling during exacerbations. However, the introduction of a test that reflects airway or systemic inflammation in the diagnosis of exacerbations might be another step forward in the management of COPD.

## Commentary article

Chronic Obstructive Pulmonary Disease (COPD) is characterized by exacerbations which are caused mainly by infections of the tracheobronchial tree or by inhalation of toxic gases [1]. During an exacerbation airway inflammation augments and a sustained worsening of patient's condition from the stable state and beyond normal day-to-day variations is observed. COPD exacerbations are a major cause of hospital admissions and frequent exacerbations are associated with increased mortality and impaired health-related quality of life [2]. Previous studies reported also that patients who experience frequently exacerbations may present an accelerating rate of lung function decline [3,4]. In this respect, the management of exacerbations by prompt diagnosis and effective treatment that reduces exacerbation frequency [5,6], should be a major goal in COPD.

However, despite the considerable progress in the understanding of the pathobiology of exacerbations and in the evaluation of their consequences, yet, there is no standardised definition of an exacerbation. The landmark study of Fletcher and Peto[7] published in seventies used the definition of "bronchial infections" (chest cold or illnesses during which phlegm production had increased). Later, the Lung Health Study[8] used the definition of "physician visits for lower respiratory illness", while in recent studies such as in the East London Study[3] and in a study performed in Greece[4], the definition of an exacerbation was based on criteria described previously by Anthonisen [9]. These criteria require either, increase of at least two major respiratory symptoms (dyspnea, sputum amount, and sputum purulence) or, increase of one major symptom in addition to at least one minor symptom (wheeze, cough, fever, nasal discharge, sore throat), for at least two consecutive days. Thus, in those previous studies, definitions were based either on patient reported symptom changes [3,4] or, on "healthcare utilisation" due to the worsening of patients condition [10,11]. However, it is not known which definition has been better.

Both these two types of definition presented certain drawbacks. Exacerbations definition, based on symptoms and signs, is limited by the fact that respiratory symptoms and signs (increased dyspnea, cough, sputum) which are used to characterize exacerbations, are not specific and can be also found in other clinical conditions. In this respect, common colds, respiratory infections or congestive cardiac failure may mimic an exacerbation in a patient with underlying COPD [12-14]. In addition, COPD patients experience chronic symptoms and mild changes in their baseline symptoms which may be undetected by the physician while many patients do not report their symptoms to physicians or they report it with a delay[15,16]. On the other hand, the "healthcare utilisation" definition (such as the need for supplementary medications (i.e. oral corticosteroids)[10,11], may be related to the access to health care services (such as the social and financial situation of the patient) which vary between countries [4,10]. Consequently, the reported average annual rates noted in most studies on COPD varied approximately between 1 to 3 depending on the definition used[4,10,11,16]. Hence, in previous recent years there has been much debate about how exactly an exacerbation should be defined [11,12].

The introduction and acceptance of a standard definition can be helpful in the clinical management of COPD patients. First, exacerbations could be detected and treated promptly. This is important since a delay in starting appropriate treatment is associated with a delay in the recovery from an exacerbation [15]. Second, frequent exacerbators [3,4] who require treatment that reduces exacerbation frequency could be identified in early stages of the disease and could be treated appropriately.

The latest GOLD executive committee recognised the necessity for a standard definition of exacerbations [1]. Although GOLD executive summary in 2001 used a general description of exacerbations based on the worsening of the clinical condition of the patient, avoiding the use a standard definition [17], GOLD has now included a definition of exacerbation in the guidelines for COPD [1]. This is an important step forward.

The current definition states that: "an exacerbation of COPD is defined as an event in the natural course of the disease characterized by a change in the patient's baseline dyspnea, cough, and/or sputum that is beyond normal day-to-day variations, is acute in onset, and may warrant a change in regular medication in a patient with underlying COPD"[13]. This definition is pragmatic and it is not in disagreement with previous definitions. Actually, the current definition compromises the different approaches for exacerbation. However, the inclusion of the "healthcare utilisation" approach (".. may warrant a change in regular medication") in the current definition may introduce in the diagnosis of exacerbation factors related to the access to health care services which may not be related to the underlying pathophysiologal process which characterizes exacerbations[14,15].

In addition, it should be noted that the aetiology of COPD exacerbations has not yet been included in the current definition. In this respect, the definition does not acknowledge the fact that many patients with COPD may suffer from additional conditions, such as community acquired pneumonia, congestive cardiac failure or pulmonary embolism that can masquerade as exacerbations but they should not be considered as causes of COPD exacerbations. In these cases the management may be challenging for physicians and mandatory for the patients. For example, patients presenting with symptoms of exacerbation may have pulmonary embolism [17] which requires anticoagulation. However, warfarin (a common oral anticoagulative therapeutic agent) interacts with steroids or with antibiotics which are required for exacerbations treatment and thus, more intensive monitoring of the anticoagulative effect is required. Therefore, inclusion of the etiologic factors of COPD exacerbations in the definition might improve further the management of these cases.

Moreover, COPD exacerbations are characterized by increased airway and systemic inflammation [18-20] and significant deterioration in lung function [13]. These fundamental aspects should be accounted in diagnosis/definition of exacerbations. This could be done by the introduction of a "laboratory" marker in the diagnosis of these acute events. Previous investigations have shown that lung function is deteriorated during exacerbations and that these tests can be performed even in patients with advanced disease [16,21]. On the other hand, there are several biomarkers in sputum and exhaled breath condensate which are significantly increased during exacerbations, reflecting the underlying airway inflammation. In addition, recent studies demonstrated that the measurement of non invasive biological markers of systemic inflammation such as plasma CRP or serum amyloid A could confirm exacerbations [22,23]. Therefore, the diagnostic value of lung function tests or of inflammatory markers in the differential diagnosis of COPD exacerbations should be further investigated in order to find the best of them for the diagnosis or the follow up of COPD exacerbations. We certainly acknowledge that the use of a test or a biomarker in the diagnosis of exacerbations requires prospective studies and meets certain difficulties related to performing lung function tests or to sampling during exacerbations [21]. However, the introduction of a diagnostic test that reflects airway or systemic inflammation in the diagnosis of exacerbations might be another step forward in the management of exacerbations for which we are all looking forward.

## Competing interests

The authors declare that they have no competing interests.

## Pre-publication history

The pre-publication history for this paper can be accessed here:


